# Gene therapy by electroporation for the treatment of chronic renal failure in companion animals

**DOI:** 10.1186/1472-6750-9-4

**Published:** 2009-01-16

**Authors:** Patricia A Brown, Angela M Bodles-Brakhop, Melissa A Pope, Ruxandra Draghia-Akli

**Affiliations:** 1 VGX Animal Health, 2700 Research Forest Drive, Suite 180, The Woodlands, Texas 77381, USA; 2 VGX Pharmaceuticals Inc., 2700 Research Forest Drive, Suite 180, The Woodlands, Texas 77381, USA

## Abstract

**Background:**

Growth hormone-releasing hormone (GHRH) plasmid-based therapy for the treatment of chronic renal failure and its complications was examined. Companion dogs (13.1 ± 0.8 years, 29.4 ± 5.01 kg) and cats (13.2 ± 0.9 years, 8.5 ± 0.37 kg) received a single 0.4 mg or 0.1 mg species-specific plasmid injection, respectively, intramuscularly followed by electroporation, and analyzed up to 75 days post-treatment; controls underwent electroporation without plasmid administration.

**Results:**

Plasmid-treated animals showed an increase in body weight (dogs 22.5% and cats 3.2%) compared to control animals, and displayed improved quality of life parameters including significant increases in appetite, activity, mentation and exercise tolerance levels. Insulin-like growth factor I (IGF-I, the downstream effector of GHRH) levels were increased in the plasmid treated animals. Hematological parameters were also significantly improved. Protein metabolism changes were observed suggesting a shift from a catabolic to an anabolic state in the treated animals. Blood urea nitrogen and creatinine did not show any significant changes suggesting maintenance of kidney function whereas the control animal's renal function deteriorated. Treated animals survived longer than control animals with 70% of dogs and 80% of cats surviving until study day 75. Only 17% and 40% of the control dogs and cats, respectively, survived to day 75.

**Conclusion:**

Improved quality of life, survival and general well-being indicate that further investigation is warranted, and show the potential of a plasmid-based therapy by electroporation in preventing and managing complications of renal insufficiency.

## Background

Renal failure and its complications, such as anemia and decreased life expectancy, can be related to primary kidney disease, such as glomerulonephritis or pyelonephritis, or are a consequence of long-term chronic diseases such as cancer, hypertension, heart failure, diabetes or severe allergic reactions [1,2]. The predicted increase in the number of people with renal failure and end-stage renal disease places an enormous burden on the healthcare provider system [3]. Strategies are therefore needed to improve the prevention, detection [4] and treatment of kidney disease.

Chronic renal failure (CRF) can affect the growth hormone releasing hormone/growth hormone/insulin-like growth factor-I (GHRH/GH/IGF-I) axis [5] which can lead to growth retardation in children and is associated with increased morbidity and mortality [6,7]. The action of GH and its mediator, IGF-I, on body composition, protein, glucose, and bone metabolism offers real therapeutic options for these patients, including the improvement of the catabolic state in adults with end-stage renal failure. Recombinant human GH has been shown to be effective in promoting growth in children of short stature with CRF both prior to and following renal transplantation [8]. Wuhl and co-investigators showed that in a few pilot studies and two placebo-controlled studies of 6 months duration, GH treatment in adults on dialysis showed clear anabolic effects resulting in a significant increase in lean body mass [9]. However, existing treatments for conditions associated with renal failure, such as anemia, wasting, immune dysfunction, or other conditions have some significant drawbacks: the daily injection routine is impractical and often associated with local or systemic adverse effects and may lead to non-compliance in patients.

The technique of electroporation (EP) is an important development for gene therapeutic approaches with the potential to treat many conditions with a single low dose of plasmid resulting in long-term effects. Previous studies using GHRH showed that plasmid therapy with EP is scalable and represents a promising approach to induce production and regulated secretion of proteins in both large animal models and in humans [10-13]. Others have also reported successful EP-mediated gene therapy or DNA vaccination in large animals [14-16]. In this current open label pilot study we have analyzed the impact of the plasmid GHRH/EP approach to treat renal failure and its complications in affected companion animals (dogs and cats) as a model for human disease. This approach has proved successful in a Phase I veterinary trial where 9 dogs with malignant melanoma were successfully treated with a xenogeneic human tyrosinase DNA vaccination delivered by the Biojector 2000 jet delivery device [17]. Furthermore, the use of companion animals provides an important bridge between preclinical studies and clinical trials while providing important information for both veterinarians and researchers involved in the study of human disease and treatments. Our results show that this gene therapeutic approach by EP can treat some of the complications of kidney failure, while increasing the well being, quality of life and life expectancy of CRF animals.

## Results

Sixty companion animals were enrolled in this pilot GHRH-study (30 cats and 30 dogs); an additional 27 were enrolled as control animals (15 cats and 12 dogs) (Table 1). No adverse effects of the plasmid administration were noted by the owners or the veterinarians caring for the animals. As this was a pilot study enlisting chronically ill companion animals, data at all time points was not available or collected due to owner compliance, especially for control animals, thus a statistical analysis was not performed for some of the parameters measured. The number of animals analyzed at each data point is specified in the cases where data from all animals was not available.

**Table 1:** Dog and cat breeds and age at enrollment.

**Treated**	**Breed Dog**	**Age at enrollment (yrs)**	**Weight at enrollment (lbs)**	**Breed Cat**	**Age at enrollment (yrs)**	**Weight at enrollment (lbs)**
**T1**	Poodle	10.9	9.5	DSH	13.9	8.5
**T2**	Shih Tzu	6.2	6.62	Calico	17.8	9.75
**T3**	Airedale	8.9	65	DMH	15.0	6
**T4**	Terrier Mix	18.0	11.31	DMH	15.1	13.41
**T5**	Bulldog	1.6	66	Bengal	9.8	9.75
**T6**	Greyhound	11.9	62	DSH	13.1	7.5
**T7**	Poodle	15.7	6.25	DSH	17.9	6
**T8**	Mixed	17.1	18.4	DSH	1.0	9
**T9**	Labrador	17.2	48.2	DSH	17.1	6
**T10**	Labrador	9.7	65.7	DLH	14.1	7.69
**T11**	Greyhound	19.1	51.3	DSH	19.1	10.53
**T12**	West Highland Terrier	15.1	22	Persian	17.1	7.1
**T13**	Yorkshire Terrier	13.7	10.8	DLH	6.4	10.5
**T14**	Yorkshire Terrier	11.4	3.31	DSH	17.1	7.4
**T15**	Bichon Frise	16.3	8.13	DSH	17.1	8.06
**T16**	Australian Shepherd	15.2	29.06	Persian	19.1	5.88
**T17**	Pomeranian	13.0	4.19	DLH	3.1	10.25
**T18**	Shih Tzu	10.3	16.56	Siamese	2.1	9.75
**T19**	Labrador	9.1	78.6	Himalayan	6.1	8
**T20**	Chihuahua	16.6	4.31	DSH	12.1	7
**T21**	Rat Terrier	15.9	10	DSH	14.9	6
**T22**	Cocker Spaniel	10.8	27.4	DSH	15.9	7.13
**T23**	Pomeranian	12.8	4.8	Siamese	14.1	9.19
**T24**	Terrier Mix	15.2	29.9	Siamese	16.9	12
**T25**	Terrier Mix	13.8	13.5	DSH	13.2	11.31
**T26**	Terrier Mix	19.3	17.75	DLH	14.5	8.31
**T27**	German Shepherd	6.9	68.4	DSH	11.2	6.4
**T28**	Shih Tzu	15.3	16	Maine Coon	15.2	7.12
**T29**	Dachshund Mix	16.9	7.3	DSH	11.2	8.88
**T30**	Labrador	10.0	101	DSH	14.2	11.25

**Average**		**13.1 ± 0.8**	**29.44 ± 5.01**		**13.2 ± 0.9**	**8.52 ± 0.37**

**Control**	**Breed Dog**	**Age at enrollment (yrs)**	**Weight at enrollment (lbs)**	**Breed Cat**	**Age at enrollment (yrs)**	**Weight at enrollment (lbs)**

**C1**	Labrador	13.92		DSH	10.87	
**C2**	Welsh Corgi	10.15	28.6	DSH	7.04	
**C3**	Maltese	11.16	5	DSH	15.76	9.3
**C4**	Shih Tzu	10.00	15.2	DSH	13.68	9.94
**C5**	Poodle	13.75		DSH	7.33	7.56
**C6**	Yorkshire/Poodle	14.54		Siamese	14.44	11.69
**C7**	Lhasa apso	15.84	15	DLH	18.59	7.12
**C8**	Chihuahua/Terrier	13.50		DLH	12.36	17
**C9**	Dalmatian	10.66		Birman	11.42	
**C10**	Lhasa apso	12.72		DLH	16.88	
**C11**	Brussels Griffon	12.79		DSH	15.82	
**C12**	Am Eskimo	15.81		DSH	14.85	
**C13**				Persian	10.31	
**C14**				DSH	16.02	
**C15**				DSH	14.57	

**Average**		**12.9 ± 0.59**	**15.95 ± 4.84**		**13.33 ± 0.87**	**10.44 ± 1.48**

Treatment with GHRH led to an increase in survival. By day 75, only 40% (n = 6 out of 15) of the control cats were still alive whereas 80% (n = 24 out of 30) of the GHRH-treated cats survived; only 17% (n = 2 out of 12) of the control dogs survived until day 75 whereas 70% (n = 21 out of 30) of the GHRH-treated dogs survived.

Body weight increased for the GHRH-treated dogs. Body weight in GHRH-treated cats remained stable with only a slight increase (Table 2). For the GHRH-treated dogs, the initial average body weight was 29.44 ± 5.01 kg (n = 27, day 0) and increased to 36.08 ± 9.07 kg (*P *< 0.05) by day 40 (n = 21), an improvement of 22.5%. The GHRH-treated cats body weight changed from 8.52 ± 0.37 kg at day 0 (n = 27) to 8.80 ± 0.58 kg at day 40 (n = 27), an improvement of 3.2%. However, the control animals exhibited a reduction in body weight over the study period. The body weight of the control cats decreased from an initial average of 10.44 ± 1.48 kg (n = 6) to 7.05 ± 0.23 kg and the control dogs weight decreased from an initial average of 15.95 ± 4.84 kg (n = 4) to 11.09 ± 3.05 kg at day 20. These control results were not statistically significant most likely due to the small number of companion animals that were able to be analyzed.

**Table 2:** Treated dog and cat average body weights during the study.

	**Treated dogs**			
	**Day 1**	**Day 20**	**Day 40**	**Day 60**
**Average weight (lbs) ± SEM**	29.44 ± 5.01	25.25 ± 4.99	36.08 ± 9.07	33.14 ± 12.01
**n**	30	22	10	6
**Ttest, *P *value**			0.046	0.166
**% Change from day 1**			22.54	12.55

	**Treated cats**			
	**Day 1**	**Day 20**	**Day 40**	**Day 60**

**Average weight (lbs) ± SEM**	8.52 ± 0.37	8.78 ± 0.44	8.80 ± 0.58	8.69 ± 0.49
**n**	30	22	14	10
**Ttest, *P *value**		0.456	0.221	0.198
**% Change from day 1**		2.97	3.24	1.99

Quality of life (QOL) parameters were not individually tabulated for control animals, but overall the attending veterinarians noted stable to worsening performance. Assessment of QOL parameters as determined by the owner's for treated dogs and cats was collected from baseline to day 60 post-treatment, and is shown in Tables 3 and 4 respectively. The overall QOL significantly improved for treated dogs compared to baseline. In particular, for treated dogs activity and exercise levels significantly improved, and appetite and mentation improved. For treated cats, the overall QOL significantly improved throughout the duration of the study, in particular, activity, exercise, mentation and appetite. Importantly, these findings regarding the QOL are similar to previously published results for dogs with spontaneous malignancies that were treated with a GHRH-expressing plasmid [13].

**Table 3:** Quality of life assessment for GHRH-treated dogs.

**Assessment**		**Day 0**	**Day 20**	**Day 40**	**Day 60**
**Overall QOL**	Average score n % change *P*-value vs. Day 0	3.08 ± 0.05 n = 26	3.43 ± 0.18* n = 23 11.63% 0.06	3.39 ± 0.20 n = 18 10.14% 0.10	**3.71 ± 0.17** **n = 17** **20.44%** **0.0005**

**Body Weight**	Average score n % change *P*-value vs. Day 0	3.0 ± 0.0 n = 28	3.0 ± 0.11 n = 19 0.0% 0.5	3.13 ± 0.20 n = 16 4.17% 0.27	3.18 ± 0.15 n = 17 5.88% 0.14
**Activity Level**	Average score n % change *P*-value vs. Day 0	2.97 ± 0.08 n = 29	**3.52 ± 0.19** **n = 23** **18.76%** **0.02**	**3.32 ± 0.19** **n = 22** **11.89%** **0.03**	**3.67 ± 0.14** **n = 18** **23.64%** **0.0001**
**Exercise Tolerance**	Average score n % change *P*-value vs. Day 0	3.07 ± 0.07 n = 29	3.21 ± 0.14 n = 23 4.84% 0.19	3.32 ± 0.19 n = 22 8.12% 0.10	**3.72 ± 0.16** **n = 18** **21.29%** **0.0003**
**Mentation**	Average score n % change *P*-value vs. Day 0	3.14 ± 0.08 n = 29	3.43 ± 0.15 n = 23 9.46% 0.10	3.14 ± 0.20 n = 21 0.16% 0.5	3.47 ± 0.18 n = 17 10.60% 0.052
**Appetite**	Average score n % change *P*-value vs. Day 0	2.98 ± 0.07 n = 29	**3.48 ± 0.19** **n = 23** **16.61%** **0.01**	3.19 ± 0.21 n = 21 6.96% 0.24	3.24 ± 0.18 n = 17 8.47% 0.07
**Thirst**	Average score n % change *P*-value vs. Day 0	3.0 ± 0.0 n = 29	3.13 ± 0.14 n = 23 4.35 0.19	3.05 ± 0.15 n = 21 1.56% 0.37	3.0 ± 0.08 n = 18 0% 0.5
**Urination Frequency**	Average score n % change *P*-value vs. Day 0	3.0 ± 0.0 n = 29	3.04 ± 0.13 n = 23 1.45% 0.37	2.95 ± 0.11 n = 21 -1.59% 0.33	3.11 ± 0.08 n = 18 3.70% 0.08
**Bowel Movement Frequency**	Average score n % change *P*-value vs. Day 0	3.04 ± 0.04 n = 28	3.09 ± 0.15 n = 23 1.69% 0.29	3.0 ± 0.12 n = 21 -1.18% 0.5	3.0 ± 0.0 n = 18 -1.18% NS
**Diarrhea Frequency**	Average score n % change *P*-value vs. Day 0	3.10 ± 0.06 n = 29	3.0 ± 0.0 n = 23 -3.33% NS	3.05 ± 0.05 n = 21 -1.80% 0.16	3.0 ± 0.0 n = 18 -3.33% 0.17
**Vomiting Frequency**	Average score n % change *P*-value vs. Day 0	3.07 ± 0.05 n = 29	2.96 ± 0.04 n = 23 -3.66% 0.04	3.05 ± 0.05 n = 21 -0.70% 0.29	3.03 ± 0.03 n = 18 -1.34% 0.09

*Abbreviations*: NS, not significant Quality of life parameters from day 0 to day 60 of study where 1 = significantly decreased, 2 = decreased, 3 = no change, 4 = increased and 5 = significantly increased. For each parameter, data are presented as average ± SEM. Percentage changes over baseline values, as well as *P *value versus baseline values is included for each parameter at each time point.

**Table 4:** Quality of life assessment for GHRH-treated cats.

**Assessment**		Day 0	Day 20	Day 40	Day 60
**Overall QOL**	Average score n % change *P*-value vs. Day 0	3.0 ± 0.05 n = 28	**3.36 ± 0.11** **n = 25** **12.0%** **0.008**	**3.43 ± 0.14** **n = 22** **14.39** **0.001**	**3.68 ± 0.19** **n = 19** **22.81%** **0.0009**

**Body Weight**	Average score n % change *P*-value vs. Day 0	3.0 ± 0.0 n = 28	3.0 ± 0.11 n = 18 0.0% 0.5	3.36 ± 0.17 n = 14 11.9% 0.03	3.18 ± 0.21 n = 17 5.88% 0.21
**Activity Level**	Average score n % change *P*-value vs. Day 0	3.07 ± 0.10 n = 28	3.27 ± 0.13 n = 26 6.44% 0.13	**3.66 ± 0.16** **n = 22** **19.13%** **0.002**	**3.68 ± 0.19** **n = 19** **19.95%** **0.005**
**Exercise Tolerance**	Average score n % change *P*-value vs. Day 0	3.0 ± 0.05 n = 28	3.23 ± 0.08 n = 26 7.69% **0.02**	3.26 ± 0.16 n = 21 8.73% 0.06	**3.61 ± 0.18** **n = 18** **20.37%** **0.002**
**Mentation**	Average score n % change *P*-value vs. Day 0	3.0 ± 0.09 n = 28	3.46 ± 0.10 n = 26 15.38% **0.002**	3.38 ± 0.15 n = 21 12.7% **0.008**	**3.45 ± 0.14** **n = 19** **14.91%** **0.005**
**Appetite**	Average score n % change *P*-value vs. Day 0	3.07 ± 0.11 n = 28	3.15 ± 0.16 n = 26 2.68% 0.26	3.23 ± 0.16 n = 22 5.07% 0.10	**3.26 ± 0.13** **n = 19** **6.24%** **0.04**
**Thirst**	Average score n % change *P*-value vs. Day 0	3.07 ± 0.07 n = 28	3.12 ± 0.08 n = 26 1.43% 0.36	3.19 ± 0.13 n = 21 3.88% 0.27	3.0 ± 0.08 n = 19 -2.33% 0.36
**Urination Frequency**	Average score n % change *P*-value vs. Day 0	3.04 ± 0.06 n = 28	3.08 ± 0.08 n = 26 1.36% 0.36	3.05 ± 0.05 n = 22 0.32% 0.5	3.0 ± 0.0 n = 19 -1.17% 0.29
**Bowel Movement Frequency**	Average score n % change *P*-value vs. Day 0	2.93 ± 0.05 n = 28	2.92 ± 0.09 n = 26 -0.19% 0.5	3.0 ± 0.0 n = 22 2.44% 0.08	3.0 ± 0.0 n = 19 2.44% 0.08
**Diarrhea Frequency**	Average score n % change *P*-value vs. Day 0	3.0 ± 0.0 n = 28	3.04 ± 0.07 n = 26 1.28% 0.29	3.0 ± 0.0 n = 22 0.0% NS	3.0 ± 0.0 n = 19 0.0% NS
**Vomiting Frequency**	Average score n % change *P*-value vs. Day 0	3.07 ± 0.05 n = 28	3.04 ± 0.07 n = 26 -1.07% 0.33	3.0 ± 0.0 n = 22 -2.33% 0.08	3.05 ± 0.05 n = 19 -0.61% 0.29

*Abbreviations*: NS, not significant Quality of life parameters from day 0 to day 60 of study where 1 = significantly decreased, 2 = decreased, 3 = no change, 4 = increased and 5 = significantly increased. For each parameter, data are presented as average ± SEM. Percentage changes over baseline values, as well as *P *value versus baseline values is included for each parameter at each time point.

**Figure 1:**
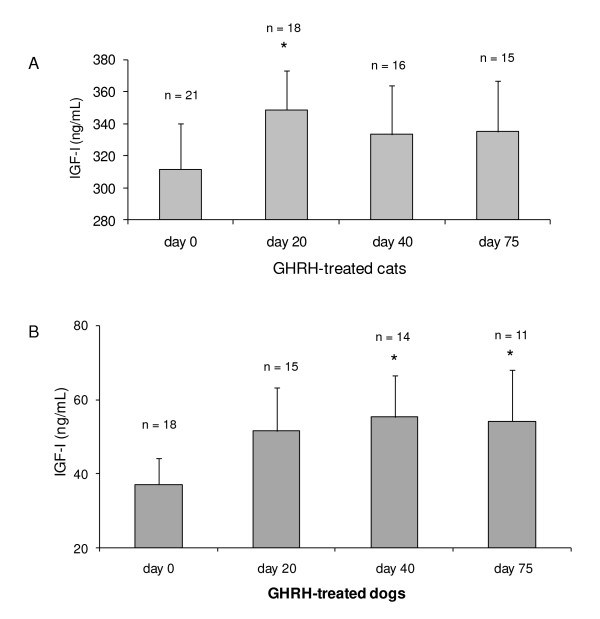
**IGF-I levels in cats and dogs with chronic renal failure treated with plasmid-mediated GHRH therapy**. The results are presented as means ± SEM. (A). 75% of GHRH-treated cats have increased IGF-I levels at 20–75 days after GHRH treatment (* *P *< 0.05). (B). 75% of GHRH-treated dogs have increased IGF-I levels at 20–75 days after GHRH treatment (* *P *< 0.05).

Cats and dogs with CRF treated with plasmid-GHRH showed an increase in their IGF-I levels compared to their baseline. Significantly increased IGF-I levels throughout the study period were seen in 72% of dogs and 65% of cats (*P *< 0.05) when compared to day 0 (Figure 1).

**Figure 2:**
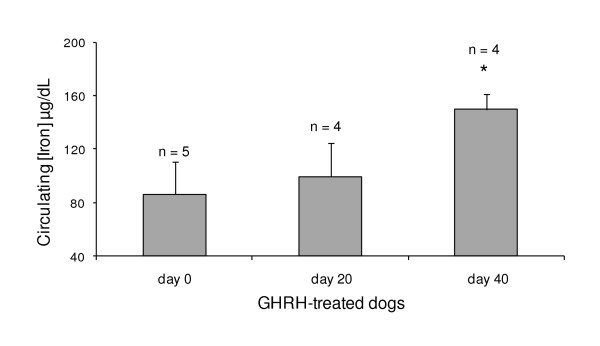
**Circulating iron concentration in GHRH-treated dogs**. The results are presented as means ± SEM. Circulating iron concentration is significantly improved in dogs treated with plasmid-mediated GHRH (where * *P *< 0.05).

In GHRH injected cats and dogs, improvements in hematological parameters compared to controls were obtained as early as twenty days after the plasmid injection. Tables 5 and 6 show hematocrit (PCV), hemoglobin (Hb), red blood cell count (RBC), and mean red cell hemoglobin (MHC) in dogs and cats with CRF treated with plasmid-mediated GHRH therapy, as well as control animals. Some disparities in response were observed between cats and dogs. This was most probably due to the heterogeneous nature of the groups, the relative moderate number of animals enrolled in the study and species differences. Percentage differences are indicated, with treated dogs showing significant changes up to 10.9% when compared to baseline levels that were below normal ranges. Hematological parameters for control dogs were stable throughout the study although a slight decrease was noted. Overall, the hematological parameters improved for the GHRH-treated animals, while remaining within or just below normal levels. Control animals displayed better hematological parameters at the time of enrollment compared to GHRH-treated animals. Increased circulating levels of iron were seen in the GHRH-treated dogs compared to baseline (Figure 2, *P *< 0.05). No significant changes in circulating levels of iron were observed in the GHRH-treated cats.

**Table 5:** Hematological parameters in plasmid-GHRH treated dogs with CRF.

**Assessment**			**Red Blood** **Cells**	**Hematocrit**	**Hemoglobin**	**Mean Red Cell Hemoglobin**
**Control**	Day 0	Average score n	6.29 ± 0.5 n = 9	39.98 ± 2.6 n = 12	13.73 ± 1.0 n = 12	23.13 ± 0.8 n = 10
	Day 20	Average score % change *P*-value vs. Day 0	6.43 ± 0.5 2.2% *P *< 0.4 n = 6	39.42 ± 1.4 -1.4% *P *< 0.2 n = 9	13.89 ± 0.8 1.1% *P *< 0.2 n = 7	22.62 ± 0.7 -2.2% ***P *< 0.05** n = 6
	Day 40	Average score % change *P*-value vs. Day 0	5.53 ± 0.4 -12% *P *< 0.3 n = 6	37.82 ± 2.2 -5.4% *P *< 0.3 n = 6	12.8 ± 1.0 -7.0% *P *< 0.4 n = 6	23.08 ± 1.1 -0.2% *P *< 0.26 n = 6
	Day 60–75	Average score % change *P*-value vs. Day 0	5.69 ± 0.2 -10.0% *P *< 0.5 n = 2	39.75 ± 1.2 -0.6% *P *< 0.5 n = 2	12.1 ± 2.2 -12.0% *P *< 0.5 n = 2	21.1 ± 3.1 -8.8% *P *< 0.19 n = 2

**Treated**	Day 0	Average score n	4.84 ± 0.3 n = 30	34.95 ± 1.9 n = 30	11.48 ± 0.6 n = 30	23.88 ± 0.2 n = 30
	Day 20	Average score % change *P*-value vs. Day 0	5.26 ± 0.3 8.6% ***P *< 0.02** n = 27	37.4 ± 1.8 7.0% *P *< 0.1 n = 27	12.74 ± 0.6 10.9% ***P *< 0.01** n = 27	24.39 ± 0.3 2.1% ***P *< 0.02** n = 27
	Day 40	Average score % change *P*-value vs. Day 0	5.13 ± 0.3 5.9% *P *< 0.09 n = 26	36.73 ± 2.1 5.1% *P *< 0.21 n = 26	12.53 ± 0.6 9.1% ***P *< 0.01** n = 26	24.58 ± 0.3 2.9% ***P *< 0.001** n = 26
	Day 60–75	Average score % change *P*-value vs. Day 0	5.19 ± 0.3 7.1% *P *< 0.15 n = 20	37.07 ± 2.2 6% *P *< 0.25 n = 20	12.69 ± 0.8 9.1% ***P *< 0.02** n = 20	24.31 ± 0.4 1.8% *P *< 0.1 n = 19

Hematological parameters are significantly improved with plasmid treatment compared to day 0. For dogs normal RBC 5.5–8.5 × 10^6^/mm^3^; normal Ht 37–55%; and normal Hb 12–18 g/dl.

**Table 6:** Hematological parameters in plasmid-GHRH treated cats with CRF.

**Assessment**			**Red Blood** **Cells**	**Hematocrit**	**Hemoglobin**	**Mean Red Cell Hemoglobin**
**Control**	Day 0	Average score n	7.19 ± .4 n = 12	31.68 ± 1.5 n = 13	11.02 ± .5 n = 12	15.44 ± .4 n = 12
	Day 20	Average score % change *P*-value vs. Day 0	7.72 ± .5 7.3% *P *< 0.16 n = 7	32.87 ± 2.0 3.8% *P *< 0.30 n = 10	10.75 ± .8 -2.0% *P *< 0.35 n = 8	14.27 ± .6 -8.0% *P *< 0.06 n = 7
	Day 40	Average score % change *P*-value vs. Day 0	7.36 ± .6 2.3% *P *< 0.41 n = 8	32.17 ± 2.0 1.6% *P *< 0.38 n = 10	10.27 ± .9 -7.0% ***P *< 0.05** n = 9	13.81 ± .7 -11.0% ***P *< 0.03** n = 8
	Day 60–75	Average score % change *P*-value vs. Day 0	7.40 ± .3 2.9% *P *< 0.47 n = 7	32.10 ± 1.1 1.3% *P *< 0.32 n = 7	10.99 ± .5 -0.0% *P *< 0.25 n = 7	14.9 ± .6 -4.0% *P *< 0.08 n = 7

**Treated**	Day 0	Average score n	6.16 ± 0.3 n = 30	27.59 ± 1.1 n = 30	9.65 ± 0.7 n = 30	14.73 ± 0.2 n = 30
	Day 20	Average score % change *P*-value vs. Day 0	6.71 ± 0.3 9.0% ***P *< 0.001** n = 29	30.46 ± 1.1 10.4% ***P *< 0.0009** n = 29	10.11 ± 0.4 4.7% *P *< 0.22 n = 29	15.22 ± 0.3 3.3% ***P *< 0.003** n = 29
	Day 40	Average score % change *P*-value vs. Day 0	6.35 ± 0.3 3.2% *P *< 0.06 n = 26	28.23 ± 1.1 2.3% *P *< 0.16 n = 26	9.65 ± 0.3 -0.07% *P *< 0.47 n = 26	15.32 ± 0.3 4.0% ***P *< 0.001** n = 26
	Day 60–75	Average score % change *P*-value vs. Day 0	6.42 ± 0.4 4.3% ***P *< 0.02** n = 19	29.18 ± 1.3 5.7% ***P *< 0.03** n = 20	9.82 ± 0.5 1.6% ***P *< 0.002** n = 19	15.46 ± 0.4 4.9% ***P *< 0.004** n = 19

Hematological parameters are significantly improved with plasmid treatment compared to day 0. For cats normal RBC 5.7–10.5 × 10^6^/mm^3^; normal Ht 38–52%; and normal Hb 9–16 g/dl.

**Figure 3:**
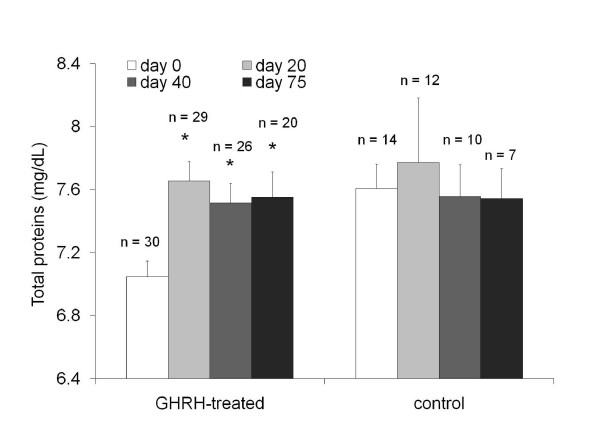
**Protein metabolism in cats with chronic renal failure treated with plasmid-mediated GHRH therapy**. The results are presented as means ± SEM. Protein metabolism is significantly improved in cats treated with plasmid-mediated GHRH (where * *P *< 0.05) whereas control cats do not show any significant differences over the time points measured.

Serum albumin and total protein values in cats and dogs with CRF were also measured. The analysis of serum albumin (normal range for cats 2.4–3.5 g/dl) for the GHRH-treated cats showed a 13% increase at 20 days post-treatment (*P *< 0.001). In addition, total protein (normal range for cats 5.3–8.5 g/dl) in GHRH-treated cats increased 9% (*P *< 0.001). These changes correlated with an improved protein metabolism, consistent with a change from a catabolic state to an anabolic state. Control cats did not significantly differ from day 0 (Figure 3). Minor increases were observed in the GHRH-treated dogs although these findings were not significant.

**Figure 4:**
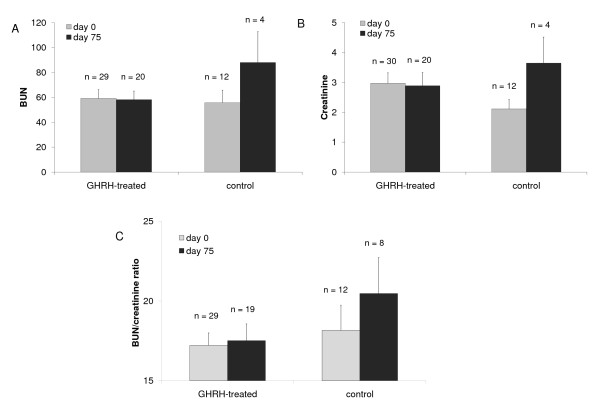
**BUN and Creatinine levels and ratio for GHRH-treated dogs and cats**. The results are presented as means ± SEM. The BUN (A) and creatinine (B) levels remained unchanged for the GHRH-treated dogs while control animals displayed an increase in BUN and creatinine at 75 days compared to day 0. (C) The BUN/creatinine ratio in cats was not significantly altered in the GHRH-treated animals but control cats showed an increase at day 75 compared to day 0.

Blood urea nitrogen (BUN) and creatinine values (indicators of renal function) were examined in dogs and cats with CRF treated with plasmid-mediated GHRH therapy. Analysis of BUN levels for the GHRH-treated dogs showed a 17% reduction, and in concert a 16% decrease in creatinine levels, whereas in control dogs BUN and creatinine levels increased by day 75 compared to day 0 (Figure 4A &4B). BUN/creatinine ratios in control cats increased from 18.15 ± 1.58 (day 0) to 20.46 ± 2.26 (day 75, *P *< 0.17), whereas the BUN/creatinine ratios in the GHRH-treated cats remained stable (Figure 4C). Overall kidney function was maintained in GHRH-treated dogs and cats whereas the controls demonstrated a trend towards deterioration of kidney function. All other parameters measured did not change significantly.

## Discussion

At present there are approximately 30 million American's affected by chronic kidney disease, with an estimated 2.2 million requiring treatment for end-stage renal disease by 2030, greatly impacting the healthcare system [18]. Malnutrition and wasting are important determinants of morbidity and mortality in patients with CRF on dialysis. Even patients with a relatively modest degree of chronic renal insufficiency are characterized by reduced lean body mass, bone mineral content, and basal energy expenditure [19]. In humans CRF has been linked to a high prevalence of anemia in an elderly population (64.9% with vs. 55.7% without CRF) with the greater risk of anemia in the presence of CRF increasing with age [20]. Most cases of CRF are associated with aging and it is one of the most common illnesses of geriatric cats and dogs [21]. Approximately one in five cats over the age of 15 yrs has CRF and it is estimated to occur 3 times more frequent in cats than dogs.

As CRF has limited therapeutic options for both humans and pets any adjuvant therapy that delays associated disorders in these patients is of high interest. Unfortunately, the lack of efficient drug therapy for supporting CRF patients significantly increases their morbidity and mortality [22]. Currently, chronic dialysis or kidney transplant are the most common forms of treatment for humans. For pets, most studies focus on nutrition and dietary changes as the only available existing therapy [23]. It has been previously shown that the administration of a GHRH-analog had also an immune-enhancing effect on healthy aging individuals through stimulation of the GHRH/GH/IGF-I axis [24]. Administration of GHRH to CRF patients may improve their quality of life by acting as an immune-enhancing agent and treating their anemia. Here we show that a one-time treatment with a plasmid expressing GHRH followed by EP can improve the outcome of companion animals with renal insufficiency.

In this open label study we have enrolled companion animals for testing of a plasmid-mediated GHRH gene therapy in a heterogeneous animal population that is more representative of a human heterogeneous population than small laboratory animals. Not only does the use of companion animals provide important relevant information for the design and follow-up of future clinical studies, but will also allow the faster development of therapies for veterinary use. It is known that GHRH stimulates the GH and further the IGF-I axis during a catabolic state, which in turn stimulates erythropoiesis [25]. IGF-I counteracts apoptosis similarly to erythropoietin, and fosters proliferation of burst- and colony-forming units-erythroid [26]. Erythropoietic agents improve survival of CRF patients, and here we have shown that a similar benefit should apply for strategies that increase synthesis and bioavailability of IGF-I. Nevertheless, it is known that erythrocytes of patients suffering from CRF are exposed to an increased activity of free radicals, which leads to a decrease of erythrocytes' stability and a lower resistance to hemolysis. The intensity of changes is often according to the stage of the disease and the efficiency of treatment. It is possible that while the GHRH-treatment improves hematological parameters and stabilizes renal function, these changes are not sufficiently maintained long-term. This short-term correction phenomenon has been previously observed in both cats and dogs with CRF on erytopoietin treatment [27,28]. A follow up study will look at the possibility of plasmid re-administration, and long-term effects of the therapy. In this study, we also found that circulating iron levels were increased in the treated patients, supporting the hypothesis that the treatment may increase iron absorption; this hypothesis will be tested in future studies. Iron deficiency is often seen in CRF and is a common cause of anemia [29]; therefore treatment with GHRH may increase iron levels without the adverse effects of parenteral iron therapy [30]. Our previous studies supported the hypothesis that an increase in GHRH will positively impact renal function, anemia, and immune dysfunction, as well as reverse wasting, and extend life expectancy of chronically ill patients. Currently, GHRH, GH or IGF-I or their functional biological equivalents are used as recombinant proteins in the treatment or management of renal failure and impaired growth that is associated with this condition. Nonetheless, the necessity for daily injections of GH [31] or other recombinant proteins is a limiting factor as the patient's (or patient's owner's) compliance is typically low and can result in sub-optimal treatment outcomes.

The technique of EP is a significant development for the field of gene therapy, given the ability to administer small amounts of plasmid in a single dose with significant effect [32,33]. Previous studies have shown that injection alone of plasmid DNA results in relatively low uptake and expression levels in comparison to injection in conjunction with EP [34], which can increase expression by up to 100 fold [35]. The use of EP along with optimization of plasmid DNA for gene delivery or DNA vaccines has become more popular as the parameters for successful delivery without tissue damage have been elucidated [36], while expression and immunogeneicity increased by 100- to 1000 fold [37].

In larger animals, we have previously shown that GHRH treatment followed by EP can dramatically improve the conditions for companion animals with cancer, resulting in an extended life span with a greatly improved quality of life [13]. Hematological parameters were also significantly improved in the GHRH-treated dogs, reversing the cancer-associated anemia. Further studies have also shown the effectiveness of GHRH treatment in combination with EP. Treatment of horses with laminitis, an arthritic-like condition, reduced inflammation, and increased survival [12]. We have previously discussed [11] and experimentally shown [38] that it is not the injection and/or the EP that determines these responses, but the effects of GHRH expression after plasmid administration. The similarity of the response across species (rats, pigs, dogs and dairy cattle) suggests that the physiologic stimulation of the GHRH axis is a fundamental component of developmental physiology. Currently, DNA vaccines or therapeutic plasmids and EP are being tested in humans in a number of Phase I or I/II clinical trials (reviewed in [39-41]).

In animal models, EP has been shown to increase the efficiency of erythropoietin (Epo) gene transfer for the treatment of CRF-associated anemia [42]. In mice and rats, treatment of severe anemia associated with CRF with Epo-gene electrotransfer, where hematocrit levels were similar to those in humans with end-stage renal disease, resulted in increased Epo and hematocrit levels [43,44]. EP also increased the transduction of optimized Epo genes reducing inter-individual and species variability [45]. We are aware of studies that have intramuscularly delivered an Epo cDNA via recombinant adeno-associated virus in monkeys and cats leading to responses against endogenous Epo [46]; nevertheless, this seems to not be the case for GHRH. We have extensively used species-specific GHRH plasmids in many models including large animals, such as pigs. In one study, we attempted to measured anti-GHRH antibodies, and no antibodies were detected against the species-specific muscle-produced GHRH [47]. Although Epo treatment is an important therapeutic strategy for renal patients with anemia, the ability to treat more than one of CRF's related complications with a single therapeutic option is of great interest. The results of this study indicated that EP of plasmid GHRH may be such a solution, and an efficient and effective way to impact many of the complications in chronically ill patients.

## Conclusion

As little as 0.1–0.4 mg of the GHRH therapeutic plasmid (equivalent to 11–13 μg/kg) delivered under the proper EP conditions in a single injection has an important biological impact by physiologically increasing IGF-I levels, that stabilizes or improves kidney function and anemia, has the ability to reverse wasting, and extend life in ailing feline or canine subjects. The treated pets display increased activity and appetite, with an increase in body weight and overall improved health and survival rate. Health-related quality of life is increasingly recognized as an important outcome in clinical research and patient care. Significant impairment in health-related quality of life is seen with renal insufficiency, due to anemia, wasting, and other complications of chronic disease or aging. Results from these studies suggest that a clinical intervention such as EP of plasmid GHRH could preserve renal function and possibly improve the negative impact of kidney disease on health-related quality of life.

## Methods

### Animals

All experiments were carried out according to NIH regulations and the National Research Councils Guide for the Care and Use of Animals, and with the specific approval of the veterinarian clinics and the attending veterinarian for each companion animal. To qualify for this study, all companion animals must have had a confirmed diagnosis of CRF from a licensed veterinarian, met eligibility requirements, and have had the owner's written consent. Patients from 11 different veterinary practices in the Houston, TX area participated in this studies. Diagnosis of renal insufficiency by the animal's veterinarian was based on physical examination, and biochemical and urinalysis parameters (underlying conditions, including possible causes of CRF, were not determined or recorded by the attending veterinarian in a majority of cases). Prior to the plasmid treatment, animals were treated with fluids (*e.g. *lactated Ringer's solution, saline, Normasol) or by specialized diet. If fitting the inclusion criteria, owners were offered to have their pets receive the GHRH-treatment or serve as controls in the study. Controls were maintained on the standard of care therapy (fluids and diet) throughout the study period. All animals were enrolled within a 6 months period (November to May). Dogs were treated with a dog-specific GHRH-expressing plasmid on average 288.86 ± 90.34 days after initial diagnosis of CRF, while cats were treated with a cat-specific GHRH-expressing plasmid 255.60 ± 61.26 days after diagnosis. The condition of inclusion in our study was an estimated survival of at least 20 days post-treatment (in order to allow for plasmid activation and expression of GHRH from the skeletal muscle) when a second blood draw could be made. The animals were weighed and bled before treatment and at days 20, 40, and 75 post-injection. At each time point, complete CBC and metabolic profiles were assessed by the same independent laboratory (Antech Diagnostics, Irvine, CA). Wellness forms were requested to be completed by owners at each visit. Body weight, activity level, exercise tolerance, mentation (attitude, alertness), appetite, thirst, frequency of urination, number of bowel movements, frequency of diarrhea, frequency of vomiting, and overall quality of life were assessed with ratings of their pets condition as significantly increased (5), increased (4), no change (3), decreased (2) or significantly decreased (1).

Case-matched control dogs (12.90 ± 0.59 years, 25.49 ± 9.04 kg) and cats (13.33 ± 0.87 years, 10.44 ± 1.48 kg) (those who's owners were offered, but did not wish to have their pets treated with the GHRH-expressing plasmid + EP, and thus not receiving plasmid but standard renal failure treatment, such as subcutaneous fluids when necessary and specialized diet) identified suitable for inclusion into the study by the attending veterinarian, were evaluated for parameters that enter into the standard of care; due to the deteriorating general state of control animals, the number of blood draws and quantity of blood per draw was maintained to a minimum in these animals in agreement with standard veterinary practice (*i.e. *hematology and biochemistry parameters were evaluated, while hormonal or other supplementary assays, such as iron or IGF-I concentrations were not performed on these group). For these parameters, baseline values were used for comparison in the GHRH-treated group.

#### Kidney Failure Dogs

Thirty dogs with kidney failure were used in this GHRH study (Table 1) with twelve additional dogs for controls. Creatinine levels had to be at least 2.5 mg/dl (normal range: 0.4–1.8 mg/dl) and blood urea nitrogen at least 35 mg/dl (normal range: 7–27 mg/dl) for the animal to be enrolled in the study. The average age of the dogs was 13.1 ± 0.56 years. The dogs were injected with 0.4 mg of a dog specific GHRH expressing plasmid, pSP-dog-GHRH.

#### Kidney Failure Cats

Thirty cats with kidney failure were used in GHRH studies (Table 1) with fifteen additional cats as controls. Creatinine levels had to be at least 3.5 mg/dl (normal range: 0.8–2.3 mg/dl) and blood urea nitrogen had to be at least 45 mg/dl (normal range: 13.4–32.5 mg/dl) for the animal to be enrolled in the study. The average age of the cats was 13.2 ± 0.66 years. The cats were injected with 0.1 mg of a cat-specific expressing plasmid pSP-cat-GHRH.

### DNA Constructs

The plasmid pSPc5-12-GHRH contains a 360-bp SPc5-12 synthetic promoter [48]. The cat and dog-GHRH cDNA was cloned in our laboratory [49]; the transgene was synthetically produced and sub-cloned into a synthetic plasmid previously described [49]. The dog-specific GHRH expressing plasmid and cat-specific GHRH expressing plasmid, for dogs and cats, respectively, were used in these studies. Plasmids were prepared by batch fermentation followed by membrane chromatography purification. The endotoxin concentration was measured with Limulus Amebocyte Lysate (LAL) kinetic chromogenic assay, and the A405 of samples were recorded with SpectraMAX PLUS 384 Microplate Reader after a period of one hour incubation at 37°C. The endotoxin levels for both preparations were less than 2 EU/mg of plasmid.

### Intramuscular injection with electroporation of plasmid DNA in canine or feline subjects with kidney failure

Endotoxin-free plasmid preparation of species-specific pSP-GHRH was diluted in sterile water for injection to 2 mg/mL, and low molecular weight poly-L-glutamate (LGS) sodium salt solution was added to 1% w/w. Dogs and cats in this study were anesthetized with isofluorane (5% for induction, 2% for maintenance). While anesthetized, plasmid was injected directly into the semitendinosus muscle. The injection was followed by constant current EP (CELLECTRA^® ^device, VGX Pharmaceuticals Inc., The Woodlands, Texas) at 0.5 Amps, 3 pulses of 52 milliseconds each, 1 second between pulses with an IM applicator consisting of 1.375 ± 0.020 inch long 21-gauge needles in a pentagonal arrangement [50]. Animals were allowed to recover from the anesthesia before rejoining their owners. Control animals underwent the EP procedure but were not injected with the GHRH-plasmid.

### Body Weight, Blood and Urine values

Animals were weighed before the plasmid injection and at three post-treatment time points post-injection/EP using the same calibrated scale. One pre-injection/EP blood draw (day 0), and three post-treatment draws (at approximately days 20, 40, 75) were performed. Blood and urine samples, obtained by standard veterinarian practice, were analyzed for biochemistry, metabolism and hormones. Complete blood count (CBC), biochemistry and hormone values for day 0 served as baseline reference for each dog or cat. Whole blood was collected in Monoject^® ^Lavender Stopper blood collection tubes with 3.0 mg EDTA (Sherwood Medical, St. Louis, MO) and submitted for CBC analysis (Antech Diagnostics, Irvine, CA). Serum was aliquoted for radioimmunoassay and biochemical analysis (Antech Diagnostics, Irvine, CA).

### Plasma IGF-I

IGF-I levels were not determined in control animals as there were too few blood samples to warrant statistical testing (as only a small number of animals survived to the end of the study); IGF-I levels were measured in treated dogs and cats throughout the study and compared to baseline values. IGF-I levels were measured by heterologous human radioimmunometric assay from collected blood samples (Diagnostic System Lab., Webster, TX). The sensitivity of the assay was 0.8 ng/ml; intra-assay and inter-assay variation was 3.4% and 4.5% respectively. All samples were analyzed in the same assay and the intra-assay variability was 4.2%.

### Statistics

A Microsoft Excel statistics analysis package was used. Values shown in the Figures are the mean ± SEM. Specific *P *values were obtained by comparison using ANOVA. *P *< 0.05 was set as the level of statistical significance.

## Abbreviations

GHRH: growth hormone-releasing hormone; GH: growth hormone; IGF-I: insulin-like growth factor-I; CRF: chronic renal failure; EP: electroporation; QOL: quality of life; PVC: hematocrit; Hb: hemoglobin; RBC: red blood cell; MHC: mean red blood cell hemoglobin.

## Authors' contributions

PB participated in the design of the study, helped with electroporation and collected samples. RD conceived of the plasmid design, the study, and participated in its design and coordination and helped to draft the manuscript. AB drafted the manuscript. MP helped collect samples and carried out the immunoassays. MP participated in the construct design. All authors read and approved the final manuscript.
